# Anorexia nervosa among teenage girls: Emerging or prevalent?

**DOI:** 10.12669/pjms.316.7617

**Published:** 2015

**Authors:** Aliya Hisam, Mahmood Ur Rahman, Syed Fawad Mashhadi

**Affiliations:** 1 Aliya Hisam, MBBS, MPH. Assistant Professor, National University of Sciences and Technology (NUST), Community Medicine Department, Army Medical College, Abid Majeed Road, Rawalpindi, Pakistan; 2 Mahmood Ur Rahman, MBBS, DPH, MPH, MSc, FCPS. Professor and Head of Department, Community Medicine Department, Army Medical College, Abid Majeed Road, Rawalpindi, Pakistan; 3 Syed Fawad Mashhadi, MBBS, MPH, MPhil, MCPS. Assistant Professor, Community Medicine Department, Army Medical College, Abid Majeed Road, Rawalpindi, Pakistan

**Keywords:** Knowledge, Practice, Anorexia Nervosa

## Abstract

**Objectives:**

To find out frequency of anorexia nervosa (AN) among teenage girls (TG) and to find out the knowledge and practice regarding anorexia nervosa among teenage girls.

**Methods:**

A cross sectional study was conducted at higher secondary public school, Rawalpindi from June 2013 till December 2013. A sample of 100 female students of the age group 13-19 years were inducted by systematic sampling technique. Mixed pretested questionnaire was filled after informed verbal consent. Data was entered and analysed using SPSS version 20.

**Results:**

Participants mean age was 15.81 ± 1.323 years. Mean weight, mean height and mean body mass index were found to be 50.34 ± 10.445 kg, 160.14 ± 7.846 cm and 19.675 ± 4.1477 kg/m^2^ respectively. Anorexia nervosa was found in 42 (42%) teenage girls while 58 (58%) were not having anorexia nervosa. Sufficient knowledge and positive practice were found to be present in 57 (57%) and 49 (49%) respectively. Statistically no significant association was found between KP and AN (p=0.73).

**Conclusion:**

Anorexia nervosa is an emerging health concern in Pakistan. Anorexia prevalent behaviour was observed in almost half of the teenage girls.

## INTRODUCTION

Psychiatric disorder having the highest mortality is Anorexia Nervosa (AN), a perplexing illness. It is characterized by low body weight, intense fear of weight gain, body image distortion and amenorrhea.^1^

Body weight and its perception play an important role in the physical and mental well-being of a person.^2^ Children and adolescent with eating disorders develop acute and chronic medical complications affecting multiple organ systems. Profound disabling medical complications occur when malnutrition occurs during normative periods of bone growth and organ development.^3^

The onset of anorexia usually occurs during adolescents with a median age of 17 years.^4^ About 0.5% to 1% of teenage girls develop anorexia nervosa (AN) in the West. Milder forms of eating disorders occur in an additional 5% to 10% of post pubertal girls.^5^ In one study, disordered eating attitudes and behaviour were present in over 27% of 1739 school girls aged 12 to 18 years.^6^ There is a need for evaluation of the risk factors in detail for eating disorders.^7^

In Australia, eating disorders are estimated to affect approximately 9% of the population. Eating Disorder Not Otherwise Specified (EDNOS) may account for up to an additional 5% of the population. Up to 20% of females may have undiagnosed eating disorder.^8^ Females with diabetes and anorexia nervosa are at 15.7% higher risk of mortality than females with diabetes alone.^8^

In a meta-analytic study, 5.9% (178 deaths in 3,006 subjects) was the crude mortality rate due to all causes of death for subjects with anorexia.^9^ Eighty-three percent of subjects with AN have at least one lifetime diagnosis of an anxiety disorder.^10^ Affected individuals can experience nutritional and hormonal problems that negatively impact bone density.^11^

Anorexia can lead to a lot of secondary diseases and can hinder normal mental and physical growth of future generations according to many studies.^8^-^11^ Unfortunately very little work has been done in Pakistan regarding the anorexia nervosa. We need to find out the frequency of anorexia nervosa among the teenage girl starting from a school setup. We have to assess the knowledge and practice of AN. Moreover we would also like to know if they are aware of the drastic consequences of anorexia nervosa and the health hazards which they are most likely to contract in near future. As Pakistan is facing double burden of malnutrition,^12^ there is a need to find out if there is an increasing frequency of eating disorder i.e. anorexia nervosa in our teenage girls.

## METHODS

A cross sectional study was conducted in a higher secondary public school (HSSC), Rawalpindi from June 2013 till December 2013. Using WHO sample size calculator, the sample size was calculated to be approximately 100 [with Confidence Level (CL) of 95%, anticipated population proportion (P) of 0.30 and absolute precision (d) of 0.09]. Female students were inducted by systematic random sampling technique of the age group 13-19 years of age. A sampling frame (list of the teenage girls aged 13-19 years) was obtained from the administration of the school. One hundred sample size was the requirement out of 335 so 335/100=3.3. Girls with visible physical disability and/or unwilling were excluded from the study. Informed verbal consent from the selected participants and their parents was taken before hand. Permission from the institute was also taken. The first subject from the sample was selected from the list numbering 1^st^ till 3^rd^ by simple random sampling (lottery method). Then afterwards, every 3^rd^ individual was selected from the list till the required sample size i.e. 100 was achieved. Data was collected using a mixed pretested questionnaire during their break time by a trained female 4^th^ years MBBS students of Army Medical College, Rawalpindi. Participants height and weight measurement was also taken and Body Mass Index (BMI) calculations recorded on their respective proformas.

Girls restricting liked food, having irrational fear of gaining weight, feeling of distorted body figure, amenorrhea, using laxative or inducing vomiting were categorized as having anorexia nervosa (AN-TG) if they have at least 3 or more of the above behaviour and/or symptoms.^13^ The other group having less than 3 symptoms were categorized as teenage girls not having anorexia nervosa (TG-NAN).

All the participants were then interviewed regarding their knowledge and practice of anorexia nervosa. Knowledge was considered sufficient if the teenage girls responded correctly to at least 2 questions related to AN. Practice was considered present if the TG were practicing at least 2 activities related to the operational definition of AN.

Data was entered and analysed using Statistical package for Social Sciences (SPSS) version 20. Qualitative variables like anorexia nervosa. Knowledge and practice etc were presented in the form of frequencies and percentages. Descriptive statistics was used to calculate mean and standard deviation for quantitative variables like age, height, weight and body mass index (BMI). Chi square test of significance was applied to find association between the KP and AN.

## RESULTS

Out of the of 100 teenage girls (TG) selected, mean age was found to be 15.81±1.323 years. Mean weight was 50.34±10.445 kg. Mean height was 160.14±7.846 cm. Body Mass Index of the TG was calculated to be 19.675±4.1477 kg/m^2^. Anorexia nervosa was found in 42 (42%) teenage girls while 58 (58%) were not having anorexia nervosa. Details regarding the mean age, weight, height and BMI is shown in Table-I.

**Table-I:** Teenage girls characteristics with or without Anorexia Nervosa (n=100).

Characteristics	TG-AN (Mean ± SD) (n=42)	TG-NAN (Mean ± SD) (n-58)	Total (Mean ± SD) (n-100)	p value
Age (years)	15.67+1.262	15.91+1.367	15.81+1.323	0.359
Weight (kg)	48.43+10.191	51.72+10.494	50.34+10.445	0.120
Height (cm)	157.83+5.364	161.81+8.941	160.14+7.846	0.012*
BMI (kg/m^2^)	19.408+3.7389	19.869+4.4422	19.675+4.1477	0.586

TG-NAN (teenage girls without anorexia nervosa) TG-AN (teenage girls with anorexia nervosa)

* P< 0.05; significant p-value.

TG were asked if they have fear of gaining weight, about 51 (51%) of them replied yes while 49 (49%) of them replied no. They were asked if they resist food what they like then 26 (26%) girls replied yes while 74 (74%) replied that they do not resist food which they like. TG were asked if they have missed their menstrual cycle during their weight loss period, about 20 (20%) of them had while 80 (80%) of them had not. While inquiring about laxative use or/and induced vomiting or/and enema, about 11 (11%) of them had tried one or the other but 89 (89%) of them had tried none of the three methods. TG were asked that when people say you are slim and normal, do you deny it because you think your body figure is not good and slim enough? About 50 (50%) of them replied yes we deny it while 50 (50%) of them said we do not deny it. About 42 (42%) girls were identified as TG-AN while 58 (58%) girls were identified as TG-NAN according to the responses to the above questions.

**Fig.1:**
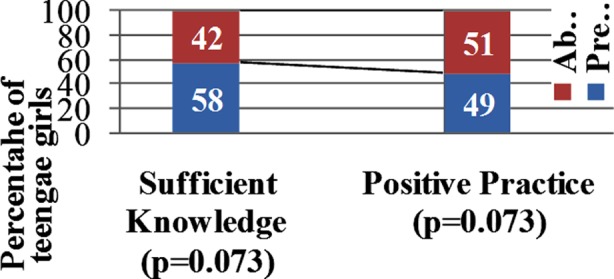
KAP among teenage girls and its association with anorexia nervosa (n=100).

Knowledge was assessed with respect to general information, causes, effect on heath and management options. Participants knowledge was sufficient in 58 (58%) while not sufficient in 42 (42%) of the teenage girls. Practice was assessed on the basis of their dietary habits, weight checking frequency, exercise intensity and frequency, use of laxatives and self inducing vomiting. Positive Practice was present in 49 (49%) and not present in 51 (51%). Association between KP and the two groups (TG-AN and TG-NAN) was statistically insignificant (p>0.05). Details are shown in Fig.1. TG knowledge regarding impending health hazards related to AN were assessed and are shown in Table-II.

**Table-II:** Knowledge regarding the impending health hazards in Teenage Girls with or without Anorexia Nervosa (n=100).

Variables	TG-AN n=42		TG-NAN n=58		p value
	Yes n (%)	No n (%)	Yes n (%)	No n (%)	
Constipation	20 (47.6)	22(52.4)	28(48.3)	30(51.7)	0.948
Bone Pains	26(61.9)	16(38.1)	32(55.2)	26(44.8)	0.501
Headache	28(66.7)	14(33.3)	40(69.0)	18(31.0)	0.808
Epigastrium Pain	15(35.7)	27(64.3)	17(29.3)	41(70.7)	0.498
Irregular menstrual cycle	21(50.0)	21(50.0)	35(60.3)	23(39.7)	0.304
Hair fall	34(81.0)	8(19.0)	41(70.7)	17(29.3)	0.242
Repeated infections	19(45.2)	23(54.8)	21(36.2)	37(63.8)	0.363
Increase sensitivity to cold	23(54.8)	19(45.2)	21(36.2)	37(63.8)	0.065
Dry mouth	17(40.5)	25(59.5)	23(39.7)	35(60.3)	0.934
Dry pale skin	22(52.4)	20(47.6)	28(48.3)	30(51.7)	0.685

TG-NAN (teenage girls without anorexia nervosa), TG-AN (teenage girls with anorexia nervosa)

## DISCUSSION

The frequency of anorexia nervosa in our adolescent group was not small enough to be ignored. Knowledge was fair enough but still practice was common regarding this eating disorder. A study conducted in The Aga Khan University Hospital, Karachi, on medical and nursing female students, showed a prevalence rate of anorexia of 21.7% among the students, which is, compared to other studies conducted in Asia, is high. But now according to our study which was conducted in a public school teenage girls showed a high percentage, as 42% of girls were found to be anorexic. This implicates that in our part of the world anorexia is more of a problem for young teenage girls.^14^ Or it could be that our operational definition of anorexia nervosa was too lenient, which could be one of our study limitations.

Another study demonstrated an increased mortality association with AN.^15^ In our study, one third of the sample was practicing vigorous exercise 3-4 times per day. A study was conducted in Karachi in the age group of 20.64 ± 1.49 years. In which the mean weight, height and BMI of the participants were found to be 53.81 ± 9.78 kg, 1.61 ± 0.06 m and 20.89 ± 3.74 kg/m^2^ respectively. In our study conducted in Rawalpindi, mean age, mean weight, height and BMI are found to be 15.81 (±1.323) years, 50.34 kg (±10.445 kg/m2), 160.14 (±7.846 cm) and 19.675 (± 4.1477 kg/m2) respectively, which is quite comparable to the study conducted at Karachi^2^ so there is need for policy makers to implement population wise strategies to assist in tackling this disorder in time.

A study in India regarding adolescent onset eating disorder (ED) was specifically written to sensitize health care professionals, pediatricians in particular about the existence of ED among adolescents.^16^ Our study can also create awareness among TG and sensitize health care professionals regarding AN emergence. In China, eating disorders prevalence was found out to be 0.90% (AN).^17^

Arcelus J et al. concluded in a systematic review that mortality rates for eating disorders not otherwise specified (EDNOS) are high especially in those with AN. The weighted annual mortality for AN was 5.10 deaths (95% CI, 3.99-6.14) per 1000 person-years.^18^ Further studies focusing on the mortality rates can be planned to assess the correct mortality pattern in our setup. Women with anorexia nervosa have a reduced bone mass due to osteoporosis.^17^ Girls with anorexia are less likely to reach their peak bone density and therefore may be at increased risk for osteoporosis and fracture throughout life.^11^ In our study, bone pain awareness may be due to the symptom appreciated by the TG-AN. The overall standardized mortality ratios (SMR) for anorexia nervosa was 6.2 (95% CI 5.5–7.0).^19^ In our study mortality rates were not calculated because it was a cross sectional study and no follow up was made.

Anorexia nervosa has caused deaths due to psychiatric, cardiac factors and nutritional deficiencies.^20^ Only a minority of people with eating disorders, are treated in mental healthcare.^21^ As in our study, frequency is almost 42% in teenage age group and if this huge percentage of TG are ignored, there could be a massive mortality and morbidity in the future women. At young age group, educational interventions must be directed. At risk people if know the consequences of eating disorder, practice regarding the eating disorders would be in less number.^22^ AN is a serious mental illness. Apart from low weight, over-exercising and food restricting are the most common history findings.^23^

Emaciation is associated with substantial medical complications i.e. compromised cardiovascular status, shifts in fluid balance, dehydration, over hydration; reduced blood levels of albumin and anemia.^1^ The high frequency of Anorexia nervosa in teenage girls as shown in our research is an alarming situation and it needs to be dealt sensibly in order to prevent the lifelong complications and mortality in AN. This study provides the basic framework for future studies and draws attention of the policy makers to take appropriate and timely action regarding this emerging grave medical condition.

## CONCLUSIONS

Anorexia nervosa is an emerging health concern in Pakistan. Anorexia nervosa prevalent behaviour was observed in almost half of the teenage girls which is way higher than other similar studies conducted in Asia. Most of the teenage girls were in fact aware of the AN impending health hazards.

## RECOMMENDATIONS

Anorexia nervosa is a treatable condition, but once a girl becomes anorexic it leaves a long term impact on her health and her offspring’s health. As these girls are going to be the child bearers of our future generations, if they indulge in such unhealthy eating activities, it would lead to a great decline in reproductive health behaviours. In light of the obtained results we would recommend the health services to collaborate with schools and halt this progression of anorexia nervosa. Awareness regarding this issue can be done by mass media communications, arranging workshops at school, college and university levels. Addition of information regarding healthy behaviour along with unhealthy eating behaviors in their curriculum can be done so as to bring progression of this morbidity to halt.
